# Supplementary material for: Collective anti-predator escape manoeuvres through optimal attack and avoidance strategies
Source: Commun Biol. 2024 Nov 27;7:1586. doi: 10.1038/s42003-024-07267-2 (PMC11603345; doi:10.1038/s42003-024-07267-2)
Supplement: Supplementary file 2 — Supplementary Information [file 42003_2024_7267_MOESM2_ESM.pdf]

Supplementary Material

Supplementary Note 1: Individual predator identities. To ascertain individual predator identities between attacks, we followed individual predators until they swam outside the video frame and could no longer be identified. This way, the predators labelled with the same ID are the same predator making multiple different attacks. However, we can not be absolutely certain about different predator IDs as the predators frequently left the frame of the video, such that it is likely that a subset of the attacks were made by the same predators at different times. Fig. 1B (in the main text) contains n=104 attacks performed by $N = 55$ (potentially) unique predator IDs. Among them, there are $N = 32$ unique predators who attacked only once, while the other $N = 23$ attacked at least twice. Out of these $N = 23$, $N = 14$ predators were identified to perform between at least $n = 3$ and $n = 5$ attacks at maximum ($N = 6$ predators did each $n = 3$ attacks, $N = 4$ predators did each $n = 4$, and another $N = 4$ predators did each $n = 5$). We subsampled $N = 8$ predators with $n = 4$ attacks each (see Fig. S1) and performed a repeated measures ANOVA to test if the individual predatory fish are behaving differently, as well as if there is a difference in the trial. There was no statistically significant difference in attack angles between individuals ($F(7,21) = 0.455$, $p = 0.855$) and also no difference in the trial number ($F(2.081,14.57) = 1.491$, $p = 0.258$). Among the 8 predators shown in Fig. S1, only 4 (I, AA, C, LL) are part of the 11 predators featured in Fig. 1D of the main text.

Fig. 1D (in the main text) is based on $n = 14$ (4 from the front, 4 from the side, 6 from the back) attacks by $N = 11$ different predators. In one back attack the fish school was not able to recover before the next attack, therefore Fig. 1E based on Fig. 1D (in the main text) incorporates only $n = 13$ attacks. These attacks were launched by $N = 10$ different predators. Each attack type has no repeated attacks by the same predator (Front: “J” (star); “F” (circle); “E” (triangle); “G” (cross). Back: “J” (star); “C” (cross); “B” (circle); “I” (dot); “D” (triangle). Side: “E” (star); “A” (circle); “D” (triangle); “K” (cross)). Between the attack types, $N = 3$ predator individuals performed repeated attacks: “J” - front and back, “E” - front and side, “D” - back and side.

Supplementary Note 2: Model choice of relative predator-prey speeds. In our modelling approach, the assumed individual predator response is given by an evasion manoeuvre away from the predator, with an additional directional bias given by the fleeing angle towards the tail of the predator. This assumption is in line with previous studies[11,21], and is also essential for the production of robust fountain-like collective responses as observed empirically. We should note that within a schooling manoeuvre, some of the early responses near the predator may correspond to what can be considered “escape responses” from a physiological perspective, as observed in previous work on schooling fishes[16,22]. These are typically fast accelerations away from a suddenly emerging threatening stimulus, with large directional variability in solitary fish[32,50] and more uniform responses in schooling fish[16]. For schools of pelagic fish being hunted by marine predators, such escape responses are likely to occur only near the predator as a last attempt to avoid capture but are unlikely to determine the overall schooling manoeuvres. Given their approximately 20 cm length (estimated mean $\pm$ std = 19.42 ± 1.92 cm) and their speed of 1.83 m/s, prey were likely to swim near their maximum swimming speed and well beyond their maximum sustainable speed (estimated to be 1.9 and 0.6 m/s, respectively, based on a 20 cm long teleost[51]), with little capacity for further acceleration. This is also a main argument for considering for simplicity a constant speed of both predator and prey. Here, we note that in a previous modelling study, extending the model towards variable prey speed, and in particular including acceleration in the escape response, did not yield any qualitative changes in the collective escape dynamics[23].

Supplementary Note 3: Effect of varying flee force strength on collective response. We explored the impact of the flee force to social force intensity ratio on prey self-organised dynamics by conducting simulation experiments with varying flee strength intensity $\mu_{flee} \in \{6,10,20,30,40\}$, while keeping the social force intensity fixed at $k = \mu_{alg} = 3$ with $\Delta\alpha^*_{flee} = 30°$ and $D_\varphi = 0.2$. The results, shown in Fig. S9 (similar to Fig. 3D in the main text), indicate that high flee intensities $\mu_{flee} > 20$ produce patterns closest to the empirical data (see Fig. 3B in the main text). This suggests that a “fountain effect” occurs primarily with sufficiently strong flee strength, while lower flee intensity results in a more “vacuole” type of collective response, where the predator is surrounded by the prey from all sides while in the middle of the prey school (Fig. S8).

Supplementary Note 4: Model sensitivity to predator attack direction, orientational noise, and prey blind angle. Our simulation results (the first row of Fig. S10) suggest that *side* and *front-side* attacks do not significantly differ from each other in terms of the closest distance attained to a predator $\langle r_{ip} \rangle$, and the same holds for the *back-side* and the *back* attacks. This implies that one can consider *front-side* with *side* attacks as a one *side*-area attack category, and *back-side* with *back* as a one *back*-area attack category in the simulation. In the main text, we focus on the extreme attack angles, referred to as back, side, and front attacks initialised at 180°, 90° and 0° respectively with additive noise in the range $(0°,30°)$. The performance for back attack relative to front and side attacks is robust at various noise levels D_φ in the absence of an additional prey blind angle (the first row of Fig. S10). We find that $D_\varphi = 0.2$ is the optimal noise intensity of fluctuations in prey orientation, where the distance to the predator $\langle r_{ip} \rangle$ is maximised in case of the back attack (i.e., facilitating prey escape) and minimised in case of the side attack (i.e., facilitating predator success). To note, $D_\varphi = 0.2$ remains optimal in terms of $\langle r_{ip} \rangle$ regardless of the prey’s blind angle within its feasible range (i.e., $\Delta\alpha_{blind} \in [15°,30°]$). For $D_\varphi = 0.2$, with an additional prey blind angle $\Delta\alpha_{blind}$

on top of the Voronoi tessellation, the optimal flee angle $\Delta\alpha^*_{flee} = 30°$ remains robust in case of the front attack and shifts to $\Delta\alpha^*_{flee} + \Delta\alpha_{blind}$ in case of back and side attacks for $\Delta\alpha_{blind} \in [15°, 30°]$) (see Fig. S10).

The interplay between the initial prey orientation, the size of its blind zone, and the predator's relative position to the prey defines whether the prey will actively flee from the predator (i.e., be a direct responder) or not. This, in turn, impacts the further social propagation of the response to the neighbours, affecting the collective response overall. In this regard, the front attack is affected the least by the presence of the blind angle, as the blind zone behind the prey does not alter the perception of the predator in front of the prey. Fig. S10 ($D_\varphi = 0$) supports this claim, as the performance for the front attack does not change regardless of the blind angle. The cases of side and back attack differ from the front one, since there the size of the blind zone affects the perception of the predator and, hence, whether the fleeing force is activated or not. In particular, the side attack case is characterised by a greater variability in detecting individuals at the edge of the school depending on their orientation, compared to an attack from behind. Moreover, the spread of the response is further impaired by the inability to detect responding prey individuals, since they may be in the blind spot of their neighbours. This results in a non-linear dependency for the distance away from the predator which the prey can build, depending on the flee angle for $\Delta\alpha_{blind} \in [45°, 60°]$, particularly in the case of $D_\varphi = 0$. The introduction of the noise on the prey orientation allows for oscillations in the position of the blind zone, creating a chance for better social propagation of the response, thereby smoothing the dependence (see $D_\varphi > 0$, $\Delta\alpha_{blind} \in [45°, 60°]$, side attack).

Supplementary Note 5: Multi-criteria analysis of simulated prey escape. The bi-objective space $(f_1, f_2) := (\langle r_{ip} \rangle, \tau)$ of the minimal averaged prey distance from a predator $\langle r_{ip} \rangle$ and collective prey recovery time τ is plotted to identify the Pareto-optimal prey fleeing angles and predator's attack directions. Concerning the effectiveness of the prey escape, a flee angle $\Delta\alpha^*_{flee}$ is Pareto optimal if it is not dominated by any other solution, i.e., there is no other $\Delta\alpha_{flee}$ for which $f_1(\Delta\alpha_{flee}) \geq f_1(\Delta\alpha^*_{flee})$ and $f_2(\Delta\alpha_{flee}) \leq f_2(\Delta\alpha^*_{flee})$, and there is a strict inequality for at least one objective. A set of all Pareto optimal solutions forms the Pareto front (PF). Fig. S12 contains the PFs of prey fleeing angles identified for each attack direction.

To identify the best compromise solution that comes as close as possible to achieving the best values for both objectives f_1 and f_2 simultaneously, we use the *ideal point* approach[52]. For each PF (depending on the attack type), we construct the ideal point I from the respective PF's *extreme solutions*, which correspond to the optimal solutions for the individual objective, such that $I^{atk} := (argmin f_2^{atk}, argmax f_1^{atk})$ within the attack type. The best compromise solution is the one on the PF with the minimal Euclidean distance ρ from the respective ideal point in the normalised objective space (i.e., the values of $f1$ and $f2$ are in the range $[0, 1]$). Table S3 shows the Pareto optimal fleeing angles $\Delta\alpha_{flee}$ for each attack type with respective Euclidean distance ρ from the ideal point I^{atk} of the corresponding attack type. That is, $\Delta\alpha_{flee} = 45°$ is the best compromise solution for the prey in back and side attacks, while $\Delta\alpha_{flee} = 60°$ is the best one for the prey in front attack.

By placing PFs of optimal prey escape angles for each attack type in the same objective space (Fig. S12), we can construct PFs of optimal attack direction from the perspective of prey and predator. We assume that the best for the prey would be to maximise the distance away from the predator (i.e., $max\ \langle r_{ip} \rangle$) and minimise their recovery time (i.e., $min\ \tau$), while the opposite is the best for the predator (i.e., $min\ \langle r_{ip} \rangle$ and $max\ \tau$). Based on the respective extreme solutions, we find the prey's ideal $E^* := (f_2^{front}(90°), f_1^{back}(30°))$ and the predator's ideal $P^* := (f_2^{back}(30°), f_1^{front}(90°))$ points (see Fig. S12). This way, the whole front-PF and the part of the back-PF (with $\Delta\alpha_{flee} \in \{30°, 45°, 60°\}$) correspond to Pareto optimal attack directions for the prey, while the whole side-PF and the whole back-PF are Pareto optimal for the predator. By computing the Euclidean distances ρ_{E^*} and ρ_{P^*} from the prey's E^* and predator's P^* ideals to the points on these PFs, we can estimate how certain attack directions, conditioned on the particular prey fleeing angle $\Delta\alpha_{flee}$, balance the trade-off between distance from the predator and the recovery time, when both objectives (f_1, f_2) are taken into consideration. Front attack with $\Delta\alpha_{flee} = 60°$ corresponds to the best attack direction for the prey (i.e., with the minimal ρ_{E^*}, Table S3). Back attack with $\Delta\alpha_{flee} = 90°$ is the closest to the predator's ideal point P^*, while side attack with $\Delta\alpha_{flee} = 45°$ is the second-closest one. However, $\Delta\alpha_{flee} = 90°$ is the extreme solution for the prey (i.e., with the minimal recovery time) without considering the second objective (i.e., distance from the predator) within the back attack ($\rho = 1$, Table S3). Therefore, conditioned on the prey response, side attack with $\Delta\alpha_{flee} = 45°$ is the best attack direction for the predator (i.e., with the second smallest ρ_{P^*}, Table S3).

Supplementary Note 6: Robustness of the collective recovery time. Figs. S15b,d,f show how estimated recovery times (i.e., $\hat{y}(\tau) = 0$) vary depending on different levels of standard deviation $\overline{\sigma}$ (std) selected in the computation of LCL_Φ (i.e., $\overline{\Phi} - 3 * \overline{\sigma}$, $\overline{\Phi} - \overline{\sigma}$, $\overline{\Phi} - 2 * \overline{\sigma}$, $\overline{\Phi} - 5 * \overline{\sigma}$). Figs. S15c,e,g show how the estimated recovery times, depending on different threshold values, relate to the value of polarisation of the prey. As we can see from Figs. S15c,e,g, there is no qualitative difference depending on the std threshold, particularly between the ones below $3 * \overline{\sigma}$, as they all quantitative correspond to high levels of polarisations $\Phi \geq 0.9$, which is indicative of an ordered group state. In other words, the results change qualitatively at the point below the defined threshold by LCL_Φ with $3 * \overline{\sigma}$. Also, Figs. S15c,e,g illustrate that choice of $3 * \overline{\sigma}$ is more robust to small perturbations in the signal compared to $5 * \overline{\sigma}$.

Table S1. Metadata on 14 analysed attack events with "fountain" escapes, describing the lowest polarisation value Φ with respective time (iter of min Φ), the lowest convexity value C of the prey school during the fountain manoeuvre with respective time (iter of min C), the start and the end of the "fountain effect", time shift introduced for Fig. 1D in the main text, and prey recovery time τ. To align the instances within the attack types by the lowest polarisation value (as in Fig. 1D of the main text), one has to add "time shift" to t^{fnt}_{start}, t^{fnt}_{end}, iter of min Φ and C, where 1 iter equals 0.01 s. The order of the attack IDs corresponds to the lexicographical order of the footage instances in Figs. S2–S4.

attack type	id	video	min Φ	iter of min Φ	min C	iter of min C	t^{fnt}_{start}	t^{fnt}_{end}	time shift	recovery time τ
front	23	DJI_3_f3943	0.195	10	0.799	8	2	6	2	10
	25	DJI_3_f4571	0.033	12	0.91	6	3	9	0	8
	15	DJI_2_f4782	0.305	10	0.757	6	1	7	2	7
	20	DJI_3_f10838	0.42	16	0.651	5	1	7	2	11
back	3	DJI_1_f1519	0.193	11	0.666	15	8	14	0	8
	7	DJI_1_f4587	0.411	6	0.737	8	2	8	5	11
	13	DJI_2_f3297	0.306	8	0.502	11	5	11	3	8
	19	DJI_3_f10577	0.048	7	0.739	9	4	9	4	12
	27	DJI_3_f7153	0.165	7	0.726	10	4	9	4	x
	29	DJI_3_f8437	0.29	11	0.704	13	9	13	0	5
side	0	DJI_1_f1355	0.57	5	0.82	9	4	8	0	4
	22	DJI_3_f19240	0.284	5	0.699	9	3	8	0	3
	10	DJI_2_f2586	0.496	4	0.666	7	1	8	1	3
	16	DJI_2_f4878	0.479	4	0.703	6	1	6	1	7

Table S2. Model parameters. The values represent dimensionless units.

Parameter	Symbol	Value
prey speed	v_0	1
angular diffusion	D_φ	0.2
alignment strength	μ_{alg}	3
attraction-repulsion strength	k	3
attraction-repulsion range	l	1
flee strength	μ_{flee}	50
flee range	R_{flee}	10
flee angle	$\Delta\alpha_{flee}$	$\{0°, 15°, ..., 90°\}$
predator initialisation distance	R_p	21.5
predator speed	v_p	2
number of prey agents	N	100
time step	dt	0.02
equilibration time	T_{eq}	200

Table S3. Euclidean distances from each Pareto front solution $\Delta\alpha_{flee}$ to: (1) prey ideal point within attack type (ρ), (2) prey ideal point E^* across attack types (ρ_{E^*}), and (3) predator ideal point P^* across attack types (ρ_{P^*}). The cross (x) denotes the solutions which are dominated (i.e., not Pareto optimal) with respect to the corresponding ideal point. The values of 1 indicate the extreme solutions on the Pareto front. The minimal value in each column corresponds to the optimal solution that is best balanced across both objectives (i.e., prey distance from the predator and prey recovery time after an attack).

attack type	$\Delta\alpha_{flee}$	ρ	$\rho_{E}*$	$\rho_{P}*$
back attack	30°	1	1	1
	45°	0.430	0.960	0.985
	60°	0.447	0.972	0.769
	90°	1	x	0.429
front attack	30°	1	0.850	x
	15°	0.894	0.792	x
	60°	0.732	0.782	x
	75°	0.739	0.837	x
	90°	1	1	1
side attack	30°	1	x	0.521
	45°	0.862	x	0.510
	60°	1	x	0.529

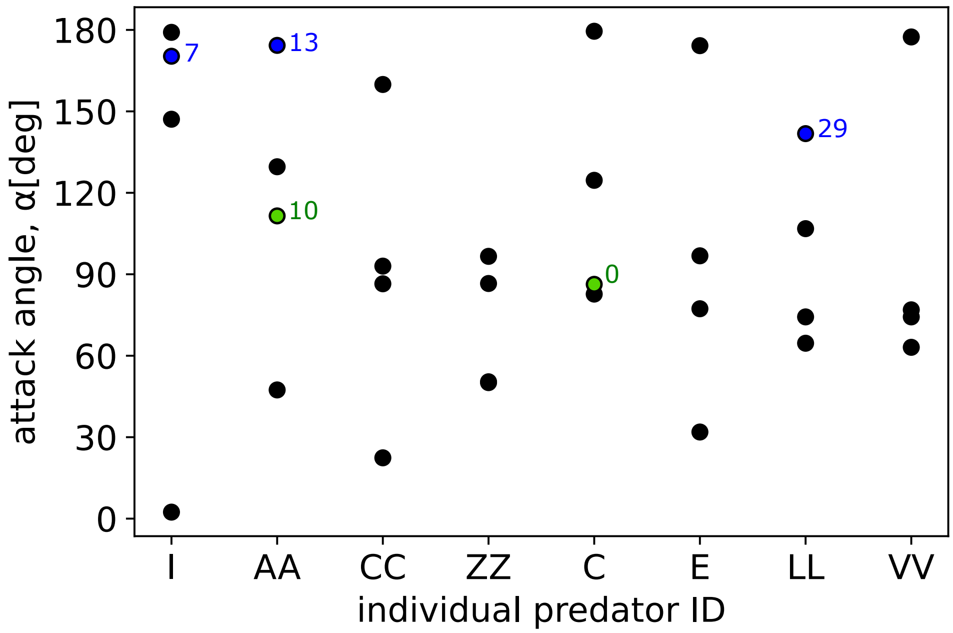

Fig. S1. Each dot shows the attack angle by an individual marlin (a subsample of 8 individual marlins with 4 attacks each), estimated by an angle between the heading direction of the prey school's centre of geometry to the predator's bill tip 3 frames $(0.1$ s) before entering the school. The attacks analysed in the main text Fig. 1D are annotated with their respective "fountain" ID numbers, as listed in Table S1, and highlighted in colour (blue for back attacks, green for side attacks). According to the repeated measures ANOVA test, there is no statistically significant difference in attack angles between individuals.

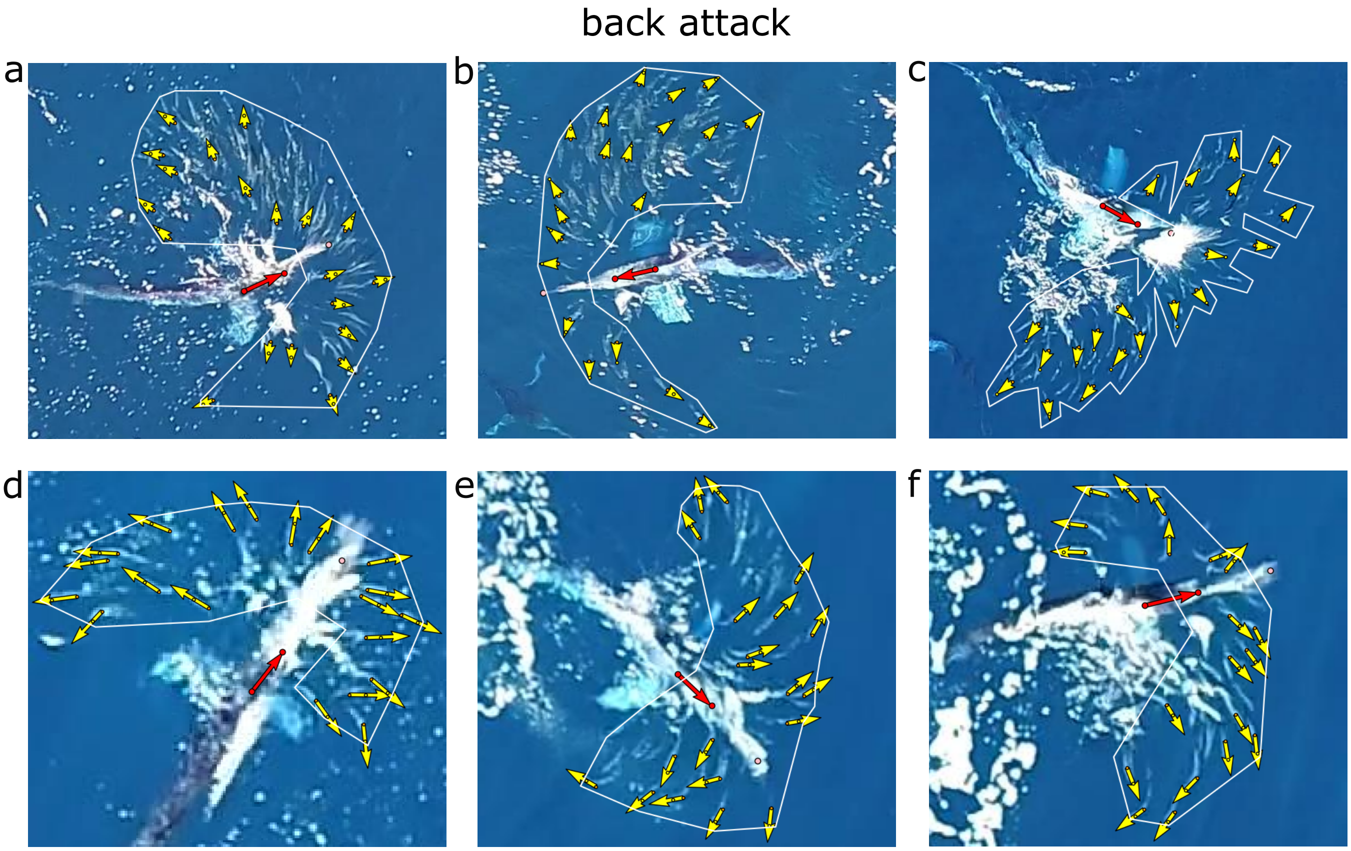

Fig. S2. Instances of the zoomed aerial footage with the "fountain effect" during an attack by a striped marlin from the back of the school. The instances correspond to the moment with the lowest convexity C of the geometry of the prey school during the attack (for more details see Table S1). The white polygon captures the borders of the prey school. Yellow arrows show the direction of motion of the annotated prey individuals, while the red arrow (connecting the marlin's fin and the head) depicts the predator's orientation. The arrows' length is independent of the speed and is normalised to be uniform within the instance. Orange and yellow dots depict the originally annotated heads and tails of the fish, respectively. The pink dot indicates the predator's bill tip.

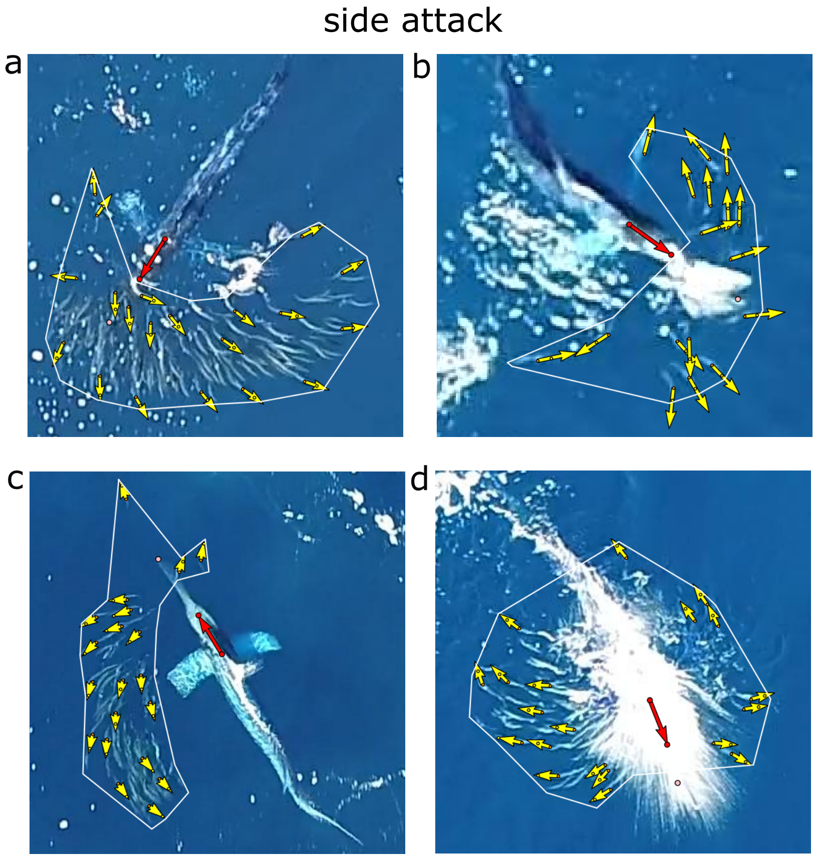

Fig. S3. Instances of the zoomed aerial footage with the "fountain effect" during an attack by a striped marlin from the side of the school. The instances correspond to the moment with the lowest convexity C of the geometry of the prey school during the attack (for more details see Table S1). The white polygon captures the borders of the prey school. Yellow arrows show the direction of motion of the annotated prey individuals, while the red arrow (connecting the marlin's fin and the head) depicts the predator's orientation. The arrows' length is independent of the speed and is normalised to be uniform within the instance. Orange and yellow dots depict the originally annotated heads and tails of the fish, respectively. The pink dot indicates the predator's bill tip.

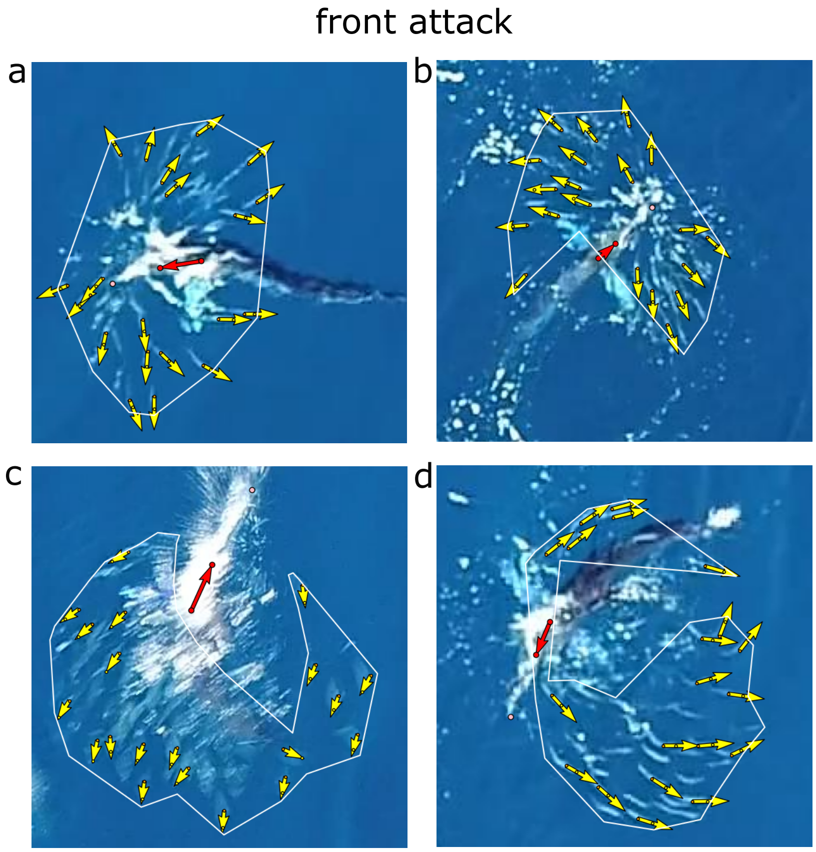

Fig. S4. Instances of the zoomed aerial footage with the “fountain effect” during an attack by a striped marlin from the front of the school. The instances correspond to the moment with the lowest convexity C of the geometry of the prey school during the attack (for more details see Table S1). The white polygon captures the borders of the prey school. Yellow arrows show the direction of motion of the annotated prey individuals, while the red arrow (connecting the marlin’s fin and the head) depicts the predator’s orientation. The arrows’ length is independent of the speed and is normalised to be uniform within the instance. Orange and yellow dots depict the originally annotated heads and tails of the fish, respectively. The pink dot indicates the predator’s bill tip.

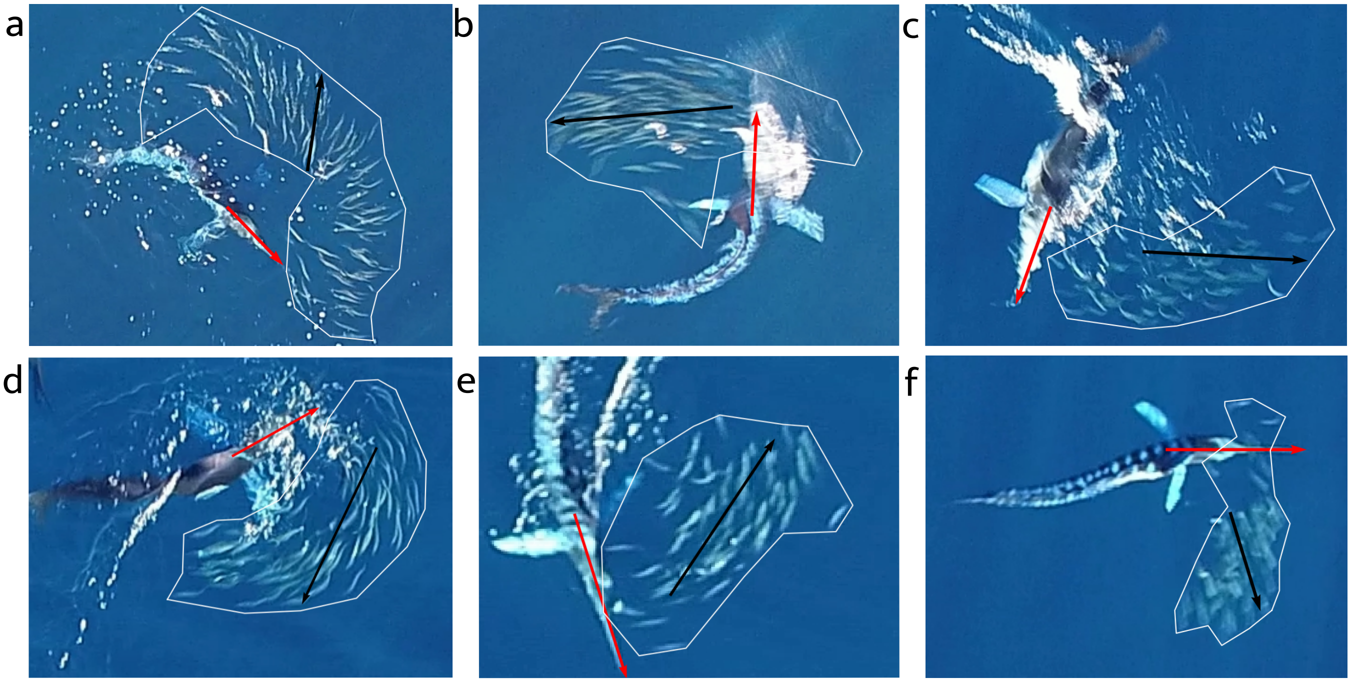

Fig. S5. Instances of the zoomed aerial footage with non-fountain escape manoeuvres during the attack by a striped marlin. The white polygon captures the borders of the prey school. The red arrow (connecting the marlin's fin and the bill tip) depicts the predator's orientation. The black arrow inside the polygon shows the general direction of motion of the whole prey school. The arrows' length is independent of the speed.

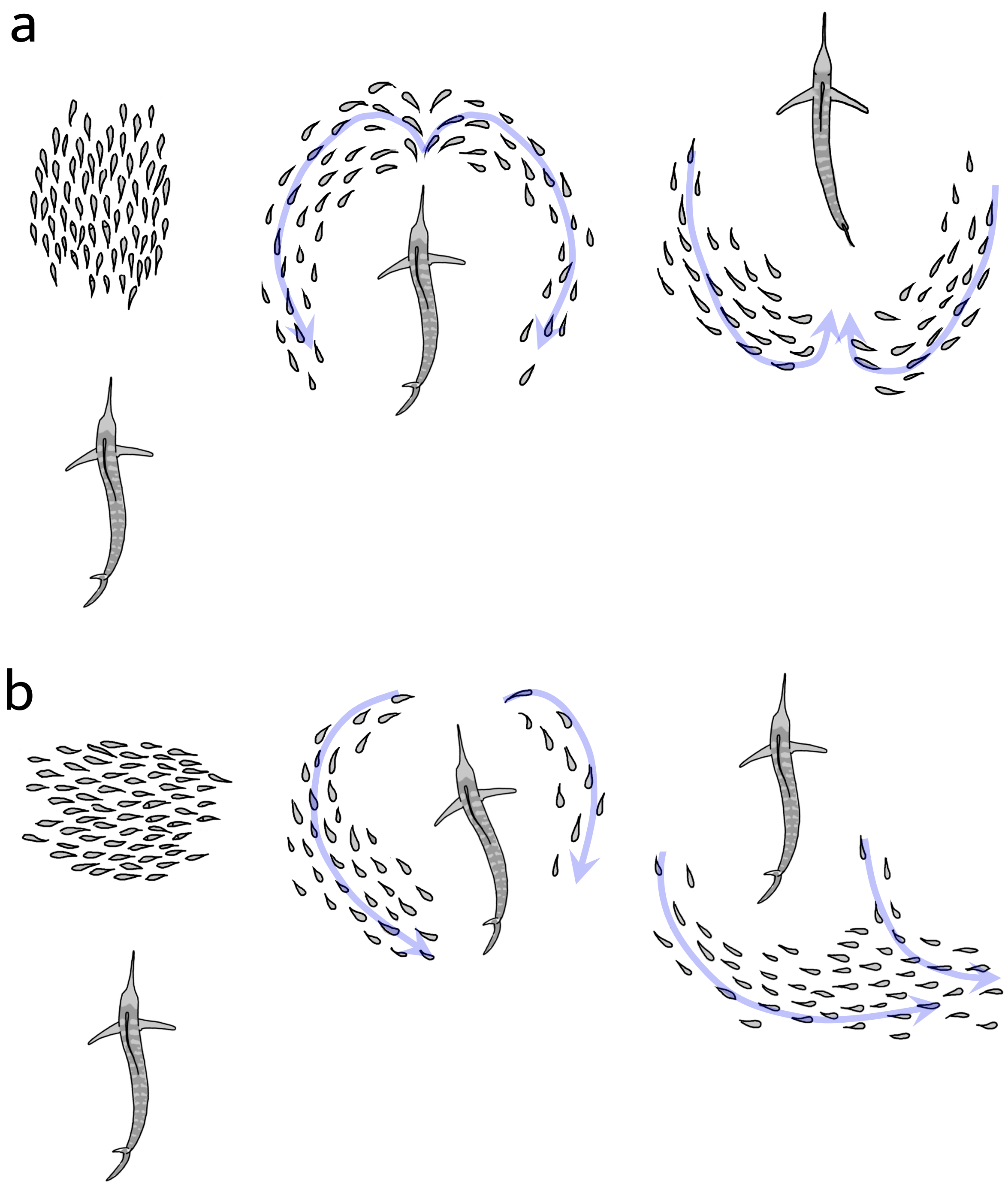

Fig. S6. Schematic illustration of the prey group splitting and re-joining behind the predator during the fountain manoeuvre, when prey is attacked from behind (a) and from the side (b). In case of the back attack (a), prey subgroups move towards each other to rejoin and re-face the same direction of motion as the predator. In the case of the side attack (b), prey subgroups realign without moving in opposite directions towards each other. Blue arrows show prey subgroups' general direction of motion during the split.

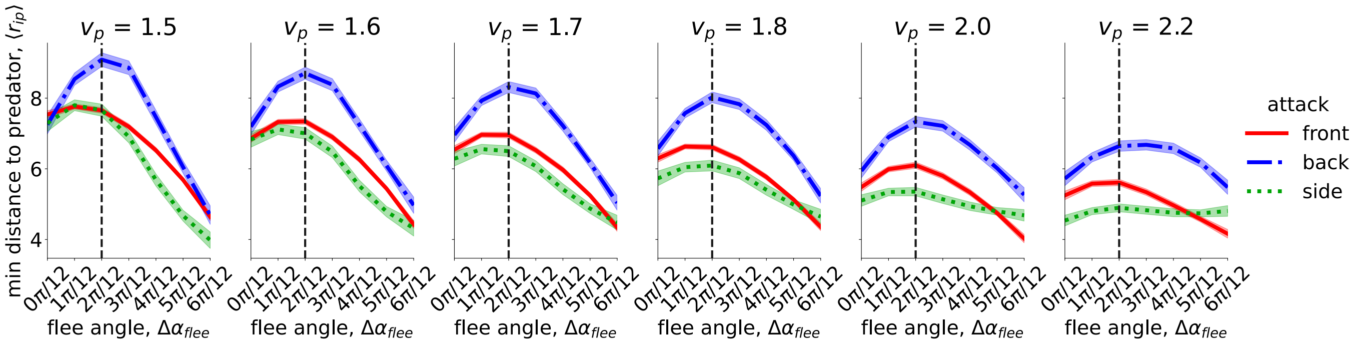

Fig. S7. The minimum distance to the predator $\langle r_{ip}\rangle := min\langle d_{ip}\rangle$ (averaged across all prey individuals i) achieved during the "fountain" window, depending on the flee angle $\Delta\alpha_{flee}$ and the predator:prey $(v_p : v_0)$ speed ratio with $v_0 := 1$. The value of the prey angular noise is set to $D_\varphi = 0.2$ and there is no additional blind angle over the Voronoi interaction network. The curves are created by local regression with shading areas of 95 % confidence interval based on 40 simulation realisations for each $\Delta\alpha_{flee}$ and v_p. The dashed vertical line shows the theoretical optimal flee angle $\Delta\alpha^*_{flee}$, as by Hall et al.[11].

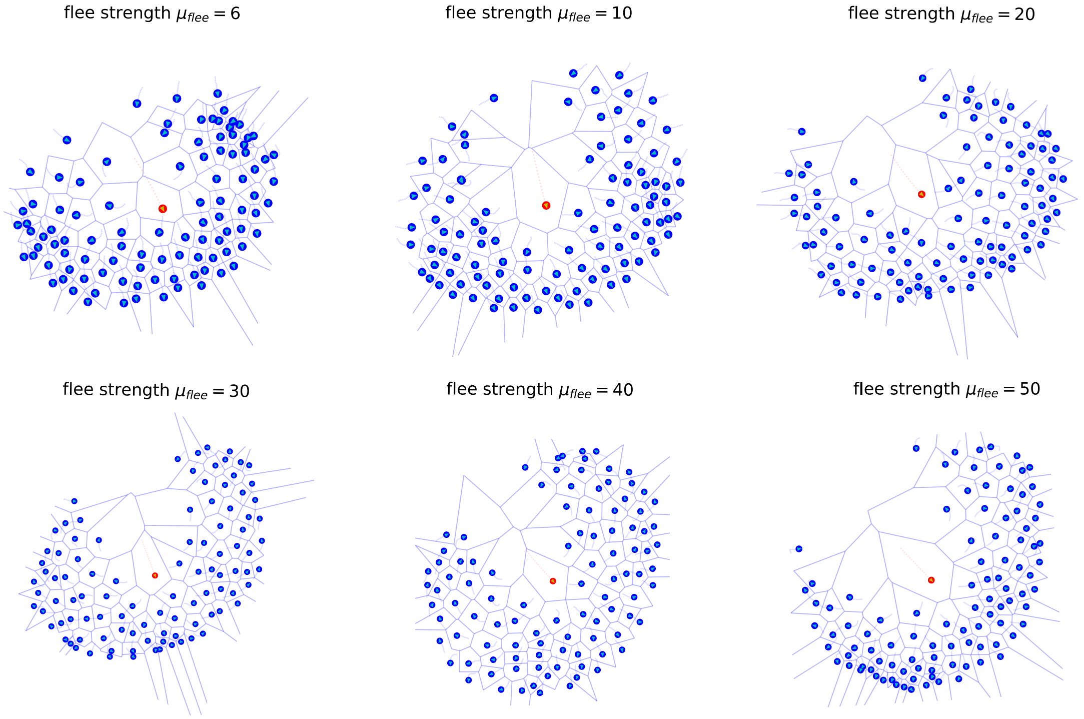

Fig. S8. Snapshots of the simulation in the middle of the attack, when the predator (red circle) attacked from the back of the prey school (blue circles), depending on the flee force intensity $\mu_{flee} \in \{6, 10, 20, 30, 40\}$, given $k = \mu_{alg} = 3$, $D_\varphi = 0.2$ and $\Delta\alpha^*_{flee} = 30^\circ$. Lower flee strength ($\mu_{flee} < 20$) results in a "vacuole" response, where prey behind the predator reunite while the predator is still within the school. In contrast, stronger flee force ($\mu_{flee} \geq 20$) leads to "fountain"-like patterns. The predator:prey speed ratio is set to $2:1$.

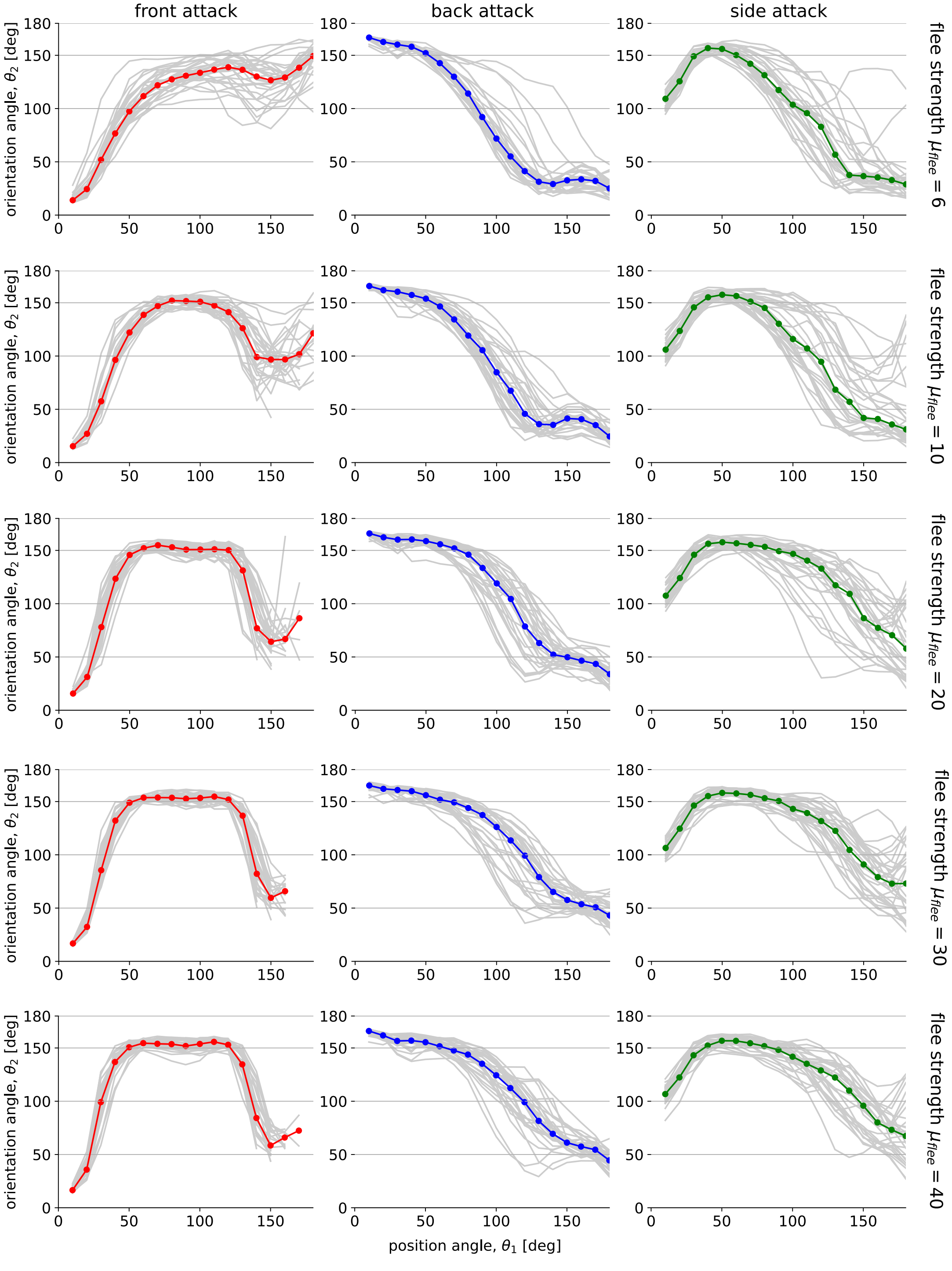

Fig. S9. Effect of varying flee force strength $\mu_{flee} \in \{6, 10, 20, 30, 40\}$ on the self-organised dynamics of the prey response in the simulation for $k = \mu_{alg} = 3$ and $D_\varphi = 0.2$. Relationship between prey's position angle relative to the predator (θ_1) and prey's orientation (swimming) angle (θ_2), given $\Delta\alpha^*_{flee} = 30°$. Each grey line represents a single simulation run, while the thick line in colour shows the average over the instances. The predator:prey speed ratio is set to $2 : 1$.

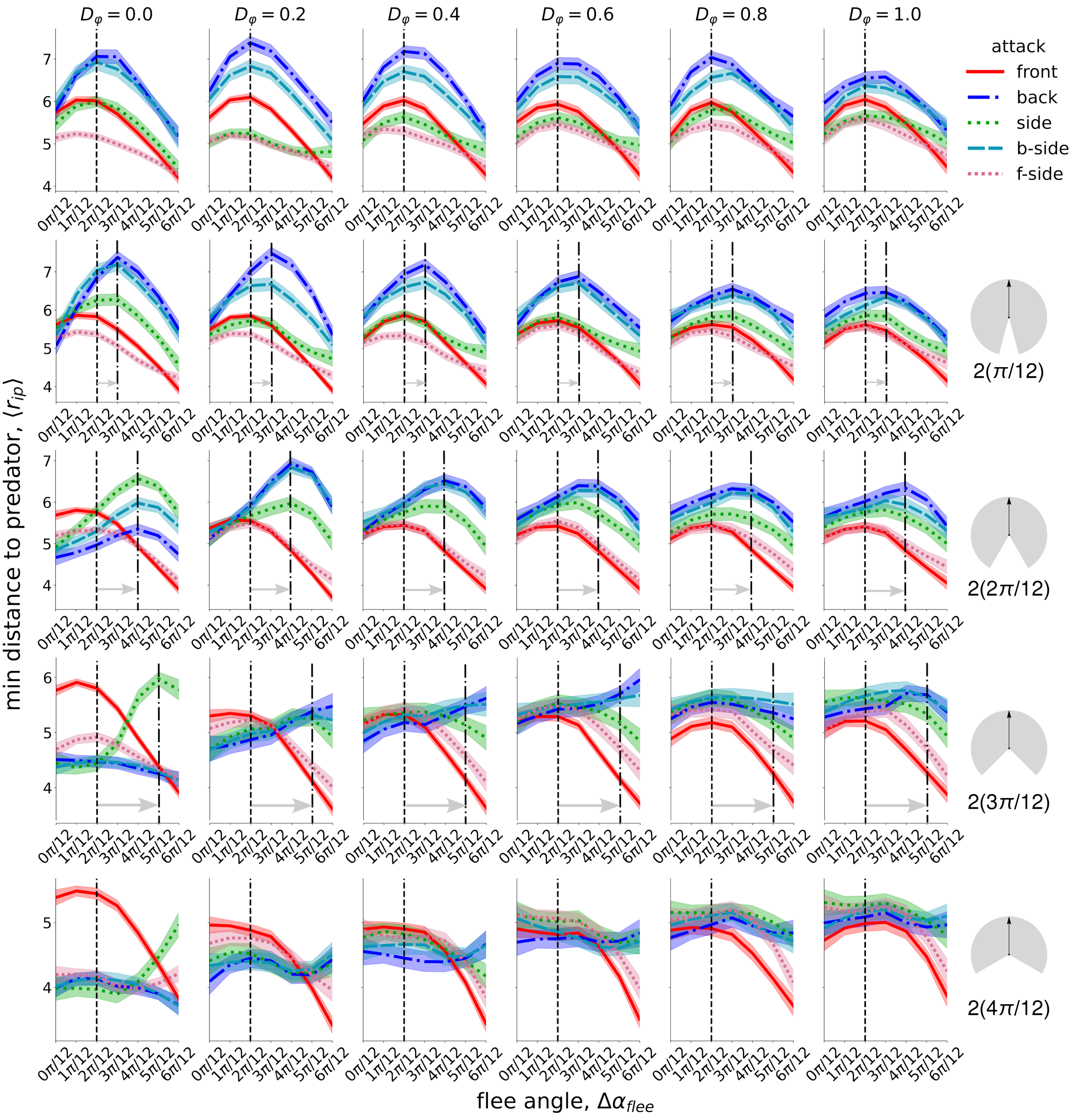

Fig. S10. The minimum distance to the predator $\langle r_{ip} \rangle := min\langle d_{ip} \rangle$ (averaged across all prey individuals i) achieved during the "fountain" window, depending on the flee angle $\Delta\alpha_{flee}$, prey orientational noise levels D_φ, and 5 attack directions. The curves are created by local regression with shading areas of 95 % confidence interval based on 40 simulation realisations for each $\Delta\alpha_{flee}$. The dashed vertical line shows the theoretical optimal flee angle $\Delta\alpha^*_{flee}$, as by Hall et al.[11]. The grey horizontal arrow indicates the shift from $\Delta\alpha^*_{flee}$ on the prey blind angle depicted by the empty sector in a grey circle on the right-hand side of each row. The first row states the results without a blind angle over the Voronoi interaction network. In teleost species, a blind zone of $10^\circ - 30^\circ$ to the rear on either side of the fish has been reported[11,53]. The predator:prey speed ratio is set to $2:1$.

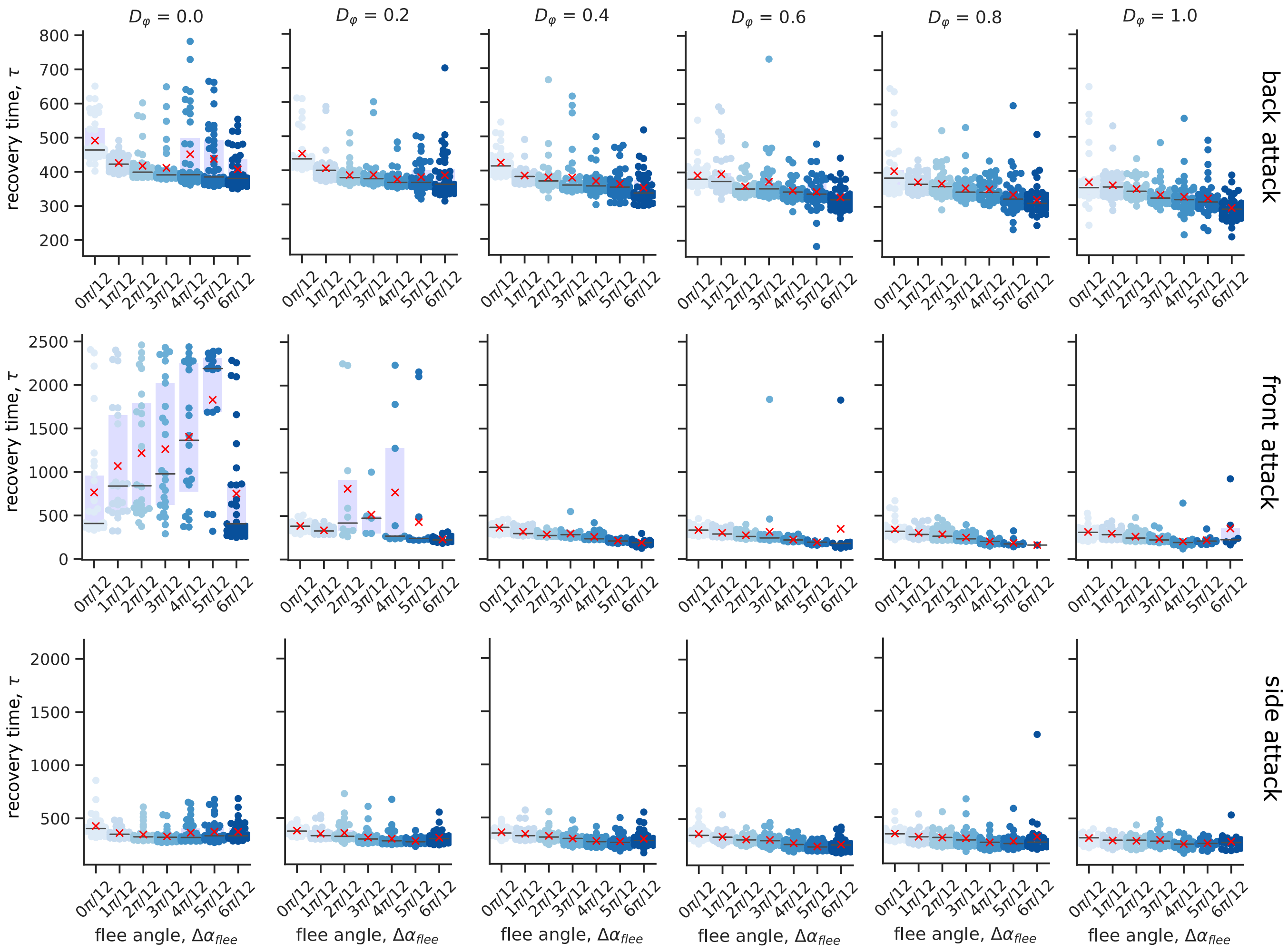

Fig. S11. Collective prey recovery time τ, depending on the prey flee angle $\Delta\alpha_{flee}$, prey orientational noise D_φ and the predator's attack direction. The cross indicates the mean and the horizontal line above the boxplot indicates the median of the 40 simulation realizations for the corresponding parameter combination. The results show that the presence of the flee angle ($\Delta\alpha_{flee} > 0^\circ$) allows for a faster collective recovery τ compared to $\Delta\alpha_{flee} = 0^\circ$, particularly, after back and side attacks. The predator:prey speed ratio is set to $2:1$.

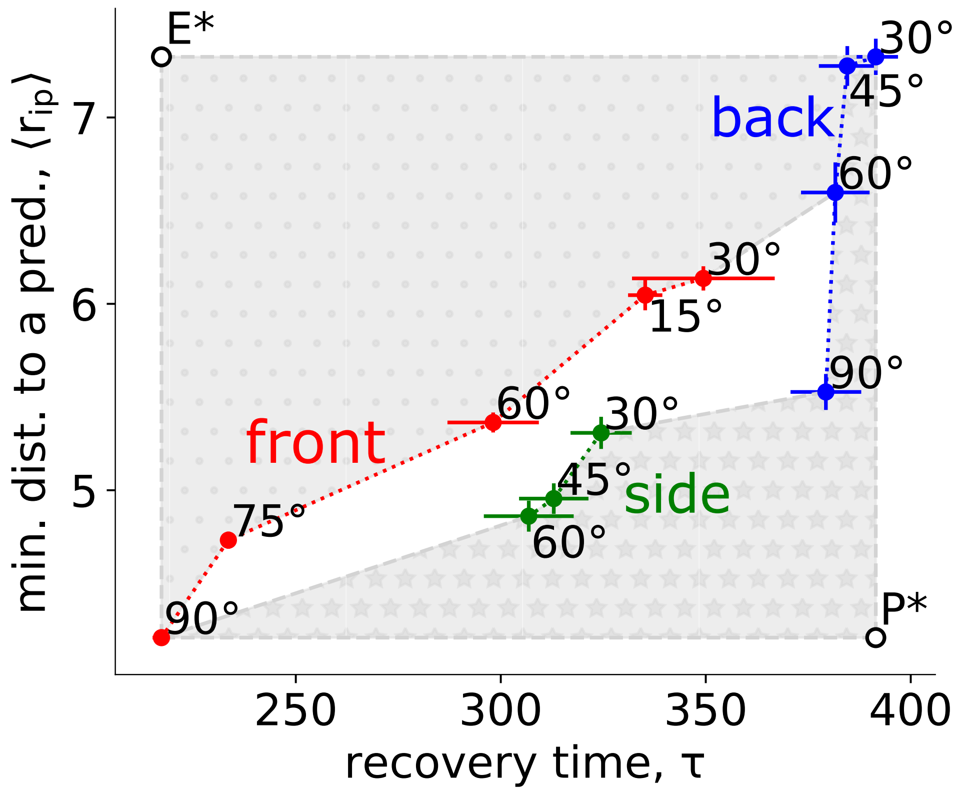

Fig. S12. Pareto fronts of attack directions, conditioned on the Pareto optimal prey escape. E^* denotes the ideal solution for the prey across all attack directions as the maximum value of minimal averaged prey distance to a predator $\langle r_{i,p} \rangle$ and the minimum value of collective recovery time τ. P^* denotes the ideal solution for the predator with the opposite goals to the prey (i.e., minimise $\langle r_{i,p} \rangle$ and maximise τ). The grey dotted area towards E^* depicts the infeasible region for the prey, with the border being the Pareto front of attack directions for the prey (front attack with $\Delta\alpha_{flee} \in \{90^\circ, 75^\circ, 60^\circ, 15^\circ, 30^\circ\}$, back attack with $\Delta\alpha_{flee} \in \{30^\circ, 45^\circ, 60^\circ\}$). The grey starred area towards P^* depicts the infeasible region for the predator, with the border being the Pareto front of attack directions for the predator (front attack with $\Delta\alpha_{flee} = 90^\circ$, side attack with $\Delta\alpha_{flee} \in \{60^\circ, 45^\circ, 30^\circ\}$, and back attack with $\Delta\alpha_{flee} \in \{90^\circ, 60^\circ, 45^\circ, 30^\circ\}$). Notably, side attack irrespective of the prey flee angle does not lie on the Pareto front of prey attack directions. Each dot represents the mean value from 40 simulation implementations, and the horizontal and vertical bars indicate the standard errors for the corresponding metric.

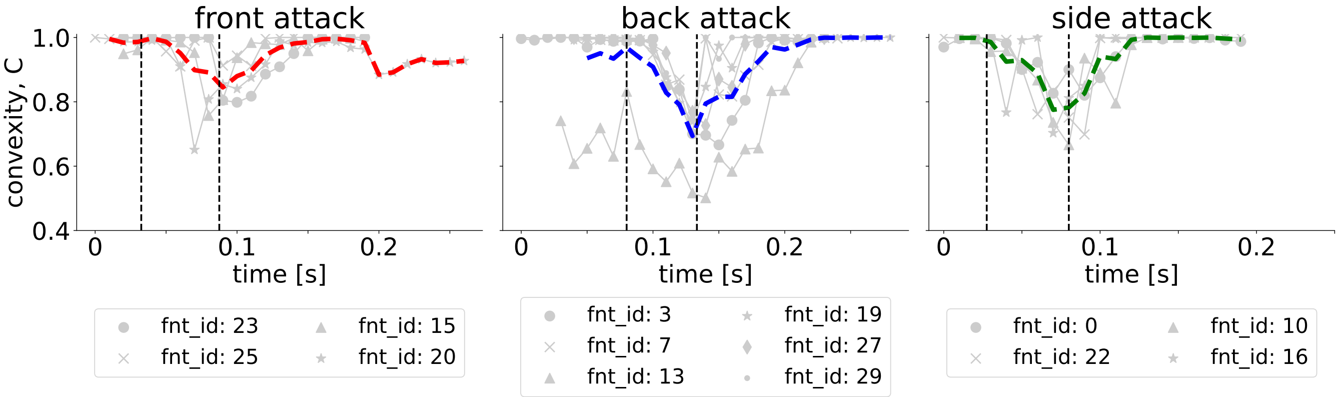

Fig. S13. Convexity C of the annotated polygon defining borders of the prey school over time in case of the fountain manoeuvre. The dashed vertical lines mark the averaged "start" and "end" times of the "fountain" over the encounters. Convexity of the school during each encounter is indicated by a grey line, while the thick line in colour shows the averaged C over the encounters of the respective attack type. Each marker corresponds to a particular fountain manoeuvre (id) and is consistent with the markers in Fig. 1D in the main text.

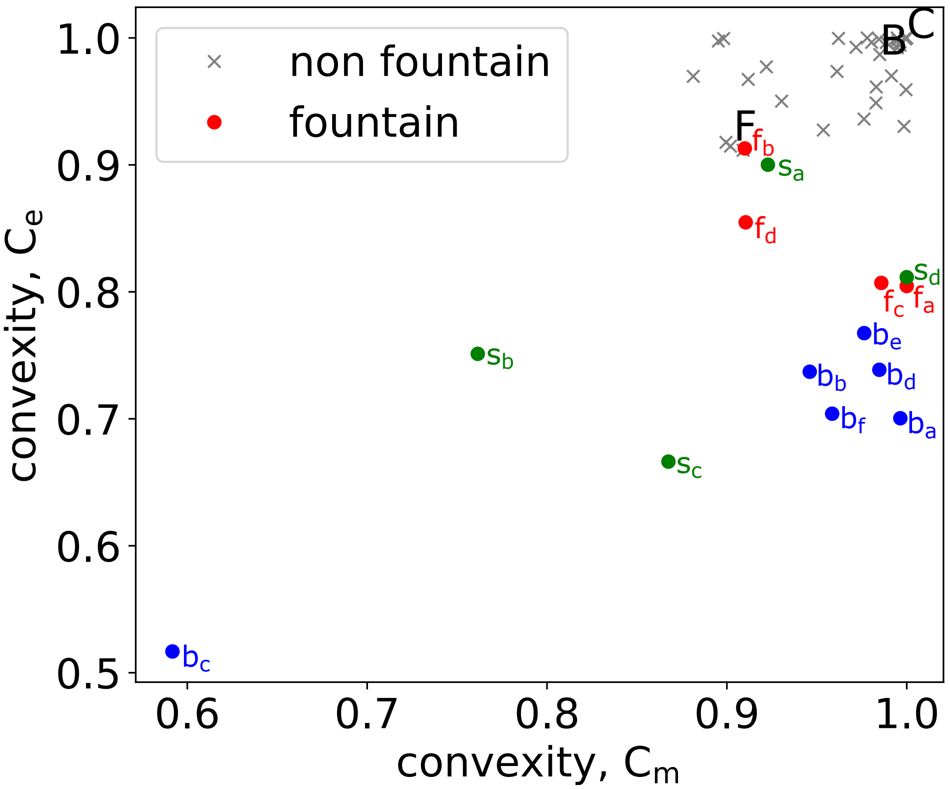

Fig. S14. Each dot shows the convexity of the polygon defining the borders of the prey school geometry in the middle C_m and in the end C_e of an attack over annotated $n = 37$ non-fountain (crosses) and $n = 14$ fountain (dots) evasions. The middle of an attack is defined as an approximate point in time between the start and the end of an attack. The start of the attack is defined as 3 frames before the predator's bill is on one level with the prey fish, such that a drawn perpendicular line to the bill's tip intersects at least with one sardine. The end is defined as when there is no more any fish in front of the mouth and the bill of the predator. The capital letters correspond to the non-fountain evasions in Fig. S5 and the lower case letters indicate the attack directions of the respective fountain evasions (denoted by the subscript letters) as in Figs. S2-S4. The attack directions are denoted by colors and lowercase letters, such as attack from the back (b in blue), side (s in green), and front (f in red).

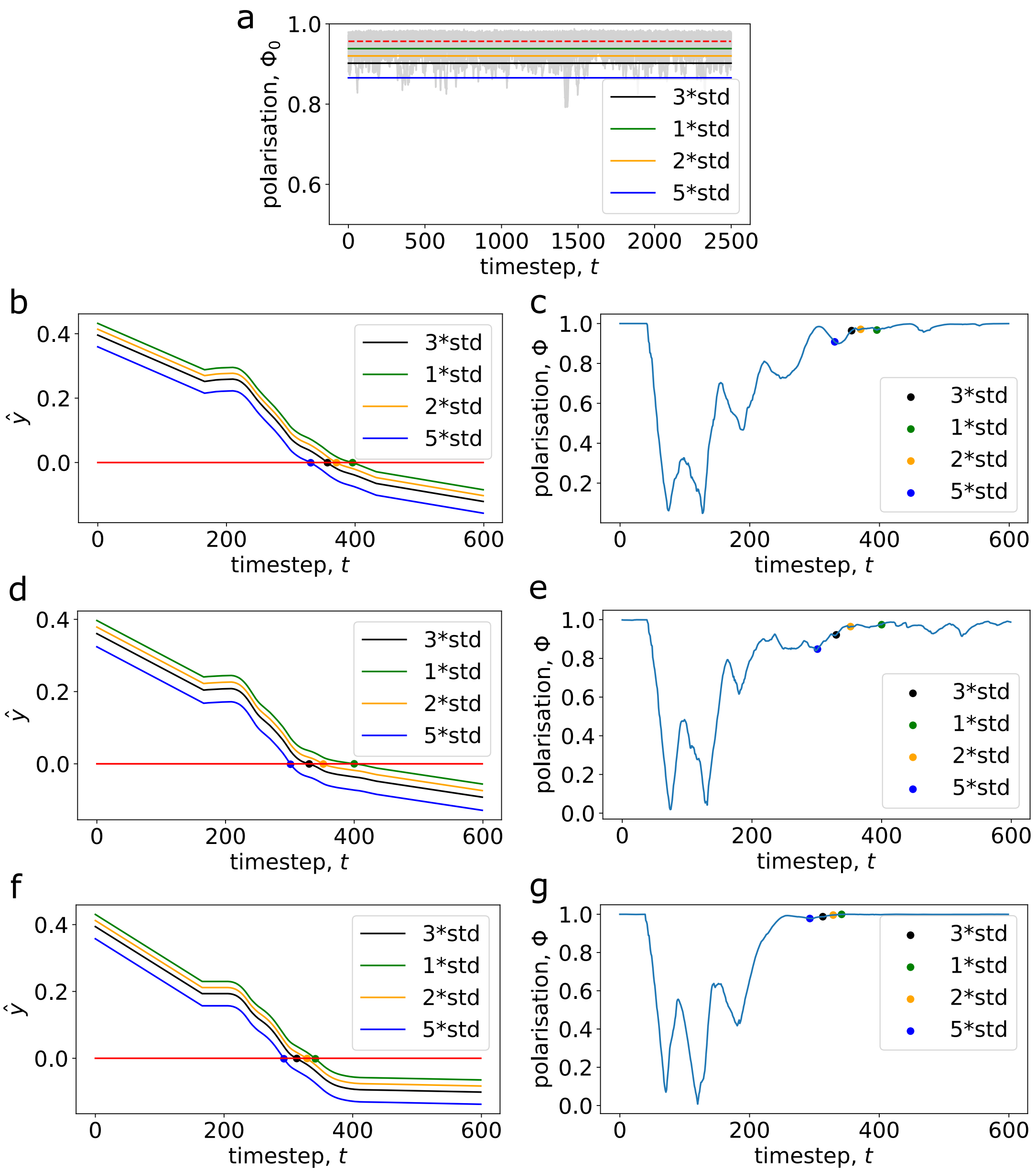

Fig. S15. (a) Time series of baseline polarization values of the non-perturbed prey group over 40 simulations (in grey) with a respective time-average mean value (dashed line in red) along with different standard deviation levels. **(b, d, f)** Instances of the smoothed time-series difference of the polarization values between the perturbed prey group and the mean value of the non-perturbed one as in **(a)**. The dots correspond to the estimated collective recovery time τ depending on the selected in **(a)** level of standard deviation ($3\bar{\sigma}$ in black, $\bar{\sigma}$ in green, $2\bar{\sigma}$ in orange, $5\bar{\sigma}$ in blue). **(c, e, f)** Instances of the time series of polarization values for the perturbed prey group with marked recovery times τ (by dots) depending on the level of selected standard deviation in **(b, d, f)** respectively.

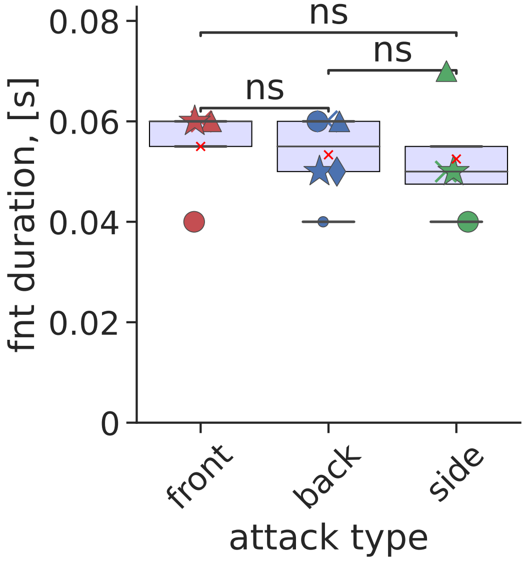

Fig. S16. Duration of the “fountain effect” depending on the attack direction (front, back, and side). According to the Kruskal-Wallis test followed by the Tukey test for multiple pairwise comparions, the results are non-significantly different (ns). The box spans from the first quartile to the third quartile, with a vertical line inside the box indicating the median value. A small red cross marks the mean value. The markers for individual events are consistent with event IDs s indicated in Fig. S7.
