# Supplementary material for: Collective anti-predator escape manoeuvres through optimal attack and avoidance strategies
Source: Commun Biol. 2024 Nov 27;7:1586. doi: 10.1038/s42003-024-07267-2 (PMC11603345; doi:10.1038/s42003-024-07267-2)
Supplement: Supplementary file 5 — Reporting Summary [file 42003_2024_7267_MOESM5_ESM.pdf]

nature portfolio

Corresponding author(s): Palina Bartashevich, COMMSBIO-24-2603-T

Last updated by author(s): Oct 31, 2024

Reporting Summary

Nature Portfolio wishes to improve the reproducibility of the work that we publish. This form provides structure for consistency and transparency in reporting. For further information on Nature Portfolio policies, see our Editorial Policies and the Editorial Policy Checklist.

Statistics

For all statistical analyses, confirm that the following items are present in the figure legend, table legend, main text, or Methods section.

n/a	Confirmed	
☐	☒	The exact sample size (*n*) for each experimental group/condition, given as a discrete number and unit of measurement
☐	☒	A statement on whether measurements were taken from distinct samples or whether the same sample was measured repeatedly
☐	☒	The statistical test(s) used AND whether they are one- or two-sided<br>*Only common tests should be described solely by name; describe more complex techniques in the Methods section.*
☒	☐	A description of all covariates tested
☐	☒	A description of any assumptions or corrections, such as tests of normality and adjustment for multiple comparisons
☐	☒	A full description of the statistical parameters including central tendency (e.g. means) or other basic estimates (e.g. regression coefficient) AND variation (e.g. standard deviation) or associated estimates of uncertainty (e.g. confidence intervals)
☐	☒	For null hypothesis testing, the test statistic (e.g. *F*, *t*, *r*) with confidence intervals, effect sizes, degrees of freedom and *P* value noted<br>*Give P values as exact values whenever suitable.*
☒	☐	For Bayesian analysis, information on the choice of priors and Markov chain Monte Carlo settings
☒	☐	For hierarchical and complex designs, identification of the appropriate level for tests and full reporting of outcomes
☒	☐	Estimates of effect sizes (e.g. Cohen's *d*, Pearson's *r*), indicating how they were calculated

Our web collection on statistics for biologists contains articles on many of the points above.

Software and code

Policy information about availability of computer code

Data collection	VirtualDub v1.10.4 (stable) https://www.virtualdub.org/; Custom code developed in Matlab and ImageJ software were used for video annotations.
Data analysis	Custom codes developed in Python v.3.8.10 were used to analyse the empirical and modeling data. The model developed in the paper is based on the published code available here: https://github.com/PaPeK/PredatorPrey. The introduced modifications to the model (code) are described in the Methods section of the paper.

For manuscripts utilizing custom algorithms or software that are central to the research but not yet described in published literature, software must be made available to editors and reviewers. We strongly encourage code deposition in a community repository (e.g. GitHub). See the Nature Portfolio guidelines for submitting code & software for further information.

Data

Policy information about availability of data

All manuscripts must include a data availability statement. This statement should provide the following information, where applicable:

- Accession codes, unique identifiers, or web links for publicly available datasets
- A description of any restrictions on data availability
- For clinical datasets or third party data, please ensure that the statement adheres to our policy

The numerical source data underlying Figs.1-4 can be found in Supplementary Data 1. The analysed videos from the footage are available on Zenodo with the

identifier https://doi.org/10.5281/zenodo.13355844.
The empirical and simulation source data underlying the footage and supporting the findings of this study are available on Zenodo under the identifiers https://zenodo.org/records/13991769 and https://zenodo.org/records/13991999, respectively.

Human research participants

Policy information about studies involving human research participants and Sex and Gender in Research.

Reporting on sex and gender	NA
Population characteristics	NA
Recruitment	NA
Ethics oversight	NA

Note that full information on the approval of the study protocol must also be provided in the manuscript.

Field-specific reporting

Please select the one below that is the best fit for your research. If you are not sure, read the appropriate sections before making your selection.

☐ Life sciences ☐ Behavioural & social sciences ☒ Ecological, evolutionary & environmental sciences

For a reference copy of the document with all sections, see nature.com/documents/nr-reporting-summary-flat.pdf

Ecological, evolutionary & environmental sciences study design

All studies must disclose on these points even when the disclosure is negative.

Study description	Quantification of schooling prey (Pacific sardines, Sardinops sagax caerulea) evasion in relation to predator (striped marlin, Kajikia audax) attacks using aerial video from drones off the coast of Baja California, Mexico. Empirical data (see research sample below) of prey evasion and predator attack angle was compared to simulation results.
Research sample	From 19 minutes of video there were 136 attacks by marlin, 104 of which predator attack angle could be quantified. 67 of these attacks produced "fountain" evasions and 37 of them produced "non-fountain" evasions. Prey positions during the "fountain" evasions were from 30 attacks (see data exclusion conditions below). Striped marlin and sardines were chosen as the sample as they could be observed hunting at the ocean's surface. There were no manipulations and the data collected were observations of wild hunts. The sample represents the striped marlin population that aggregates off the coast of Baja California during October/November/December.
Sampling strategy	Sample size was maximized from the 19 minutes of video where the positions of predator and prey could be visualised. Collecting visual data on wild hunts in the open ocean is very rare and this is the first study to collect data on prey evasion patterns (fountain effect) from video of wild predator-prey interactions. The attack and evasion patterns of striped marlin and prey are stereotypical and this suggests that the behaviours recorded in the sample represent the behaviours of the striped marlin population being studied.
Data collection	MJH, JK and FD searched for schools of sardines being predated upon by striped marlin by boat and by first locating diving birds. FD filmed the hunting behavior with unmanned aerial vehicles. PB developed and implement agent-based model and gathered the simulation data.
Timing and spatial scale	Aerial video of striped marlin and sardine school predator-prey interactions was collected opportunistically from the 10.11.2021 to the 13.11.2021 during daylight hours.
Data exclusions	Data was excluded for analysis if it was disturbed by human interference (e.g., tourists or photographers not part of the study). Data was excluded for particular analyses within the manuscript if visualizing the predator attack or prey positions was not possible due to sea conditions. Data was also excluded for some analyses in the manuscript if the predator did not move in a straight line during its attack.
Reproducibility	No experimental manipulations took place, the data is purely observational, and therefore it is impossible to reproduce exactly. The observed behaviours were superficially confirmed in other videos not used for analysis, however, they were not clear enough to accurately determine predator and prey positions. The same methods can be used each year at the same location where the predator-prey interactions can be observed (provided the predator and prey are present).
Randomization	No randomisation was required, the data collected was from purely opportunistic observations of wild predator-prey interactions.
Blinding	Blinding was not required as 'treatments ' e.g. predator attack angle or prey fountain, prey non fountain were determined post-data collection.

Did the study involve field work? ☒ Yes ☐ No

Field work, collection and transport

Field conditions	Open ocean 10-30km offshore.
Location	10-30 km offshore Baja California, Mexico (N 24° 54.52-48.5', W 112° 34.46-23.52').
Access & import/export	Empirical data collection was conducted in line with the laws and legislation of Secreteria de Medio Ambiente y Recursos Naturales (SEMARNAT), Mexico and complied to the guidelines regarding the treatment of animals in behavioural research.
Disturbance	Data was not considered for analysis if it was disturbed by human interference (e.g., tourists or photographers not part of the study).

Reporting for specific materials, systems and methods

We require information from authors about some types of materials, experimental systems and methods used in many studies. Here, indicate whether each material, system or method listed is relevant to your study. If you are not sure if a list item applies to your research, read the appropriate section before selecting a response.

Materials & experimental systems

n/a	Involved in the study	
☒	☐	Antibodies
☒	☐	Eukaryotic cell lines
☒	☐	Palaeontology and archaeology
☐	☒	Animals and other organisms
☒	☐	Clinical data
☒	☐	Dual use research of concern

Methods

n/a	Involved in the study	
☒	☐	ChIP-seq
☒	☐	Flow cytometry
☒	☐	MRI-based neuroimaging

Animals and other research organisms

Policy information about studies involving animals; ARRIVE guidelines recommended for reporting animal research, and Sex and Gender in Research

Laboratory animals	NA
Wild animals	Kajikia audax and Sardinopx sagax caerula were observed in the wild. No manipulation or captures occurred. Animals were simply video recorded from a distance of 5-10m and left undisturbed so that they displayed natural behavior.
Reporting on sex	NA
Field-collected samples	The study did not contain samples collected from the field.
Ethics oversight	Empirical data collection was conducted in line with the laws and legislation of Secreteria de Medio Ambiente y Recursos Naturales (SEMARNAT), Mexico and complied to the guidelines regarding the treatment of animals in behavioural research.

Note that full information on the approval of the study protocol must also be provided in the manuscript.
